# Deeply lonely in the borderland between childhood and adulthood - Experiences of existential loneliness as narrated by adolescents

**DOI:** 10.1080/17482631.2022.2132653

**Published:** 2022-10-06

**Authors:** Tide Garnow, Pernilla Garmy, Anna-Karin Edberg, Eva-Lena Einberg

**Affiliations:** a Faculty of Health Sciences, Kristianstad University, Kristianstad, Sweden; b WHO-CC Clinical Health Promotion Centre, Lund University, Malmö, Sweden

**Keywords:** Existential loneliness, adolescents, suffering, existential health, qualitative study, narrative interviews, content analysis

## Abstract

**Background:**

Adolescence is associated with different feelings and experiences that can negatively affect adolescents’ health and well-being. In the transition between childhood and adulthood, experiences of loneliness are common. A deep form of loneliness is described as existential loneliness. Studies among adults have shown that existential loneliness often arises in connection with transitions and is related to suffering, but may lead to positive experiences when acknowledged and addressed. Therefore, the aim of this study was to explore adolescents’ experiences of existential loneliness.

**Methods:**

This was an exploratory-descriptive qualitative study based on narrative interviews with 16 adolescents (median age 17.5). The data were analysed inductively using qualitative content analysis.

**Findings:**

Existential loneliness among adolescents was related to experiences of social exclusion and “in-betweenness”. To alleviate their suffering, the adolescents tried to avoid burdensome feelings and thoughts, and they chose between sharing or not sharing their inner lives with someone else.

**Conclusions:**

It is important to support adolescents’ sense of belonging, and they may need assistance in dealing with existential loneliness, as well as with finding constructive and healthy ways of recuperating from the suffering. Professionals need knowledge of existential loneliness to promote adolescents’ existential health and well-being.

## Introduction

Being young and being in the borderland between childhood and adulthood is associated with different, often contradictory, emotions and experiences that can evoke thoughts related to meaning and meaninglessness. Adolescence is, on the one hand, seen as the healthiest part of life (Berg Kelly & Högbom, 2014) since adolescents in general have not yet developed lifestyle diseases, and diseases related to ageing. On the other hand, adolescents experience physical and mental health problems, and several studies show that health complaints among adolescents are increasing (Inchley et al., 2020; Potrebny et al., 2017). Health complaints such as sleeping difficulties and pain are often related to mental health complaints (Potrebny et al., 2017), as well as sadness (Garnow et al., 2021) and experiences of loneliness (Hawkley & Cacioppo, 2010). Loneliness among adolescents has been shown to be related to factors such as low self-esteem, anxiety, depression and increased risk of suicide (Heinrich & Gullone, 2006), and is seen as a risk factor for adolescents’ health and well-being (Lyyra et al., 2018). Loneliness is even described as a global public health issue (Nyqvist et al., 2016; Surkalim et al., 2022), and a form of social inequality (Junttila et al., 2015). An evidence synthesis (Mansfield et al., 2021) identified three distinct but overlapping types of loneliness: social loneliness is described as a dissatisfaction with the quality of social relationships; emotional loneliness occurs due to the loss or absence of meaningful relationships; and existential loneliness is a deep form of loneliness which is seen as experiencing being disconnected from others or fundamentally separated from others and the wider world. Existential loneliness is associated with suffering and can occur even when people are around, and when having social relationships (Bolmsjö et al., 2019). It is connected to experiences of not having someone to talk to on a deep human level, which arouses negative feelings such as hopelessness, meaninglessness, and sadness. Existential loneliness has primarily been studied in disease groups (Mah, 2019; Mayers & Svartberg, 2001; Nyström, 2006; Sand, 2008), in older migrants (Chung et al., 2020; Olofsson et al., 2021), and in frail older people (Sjöberg et al., 2018, 2019). Research among adolescents has primarily focused on loneliness in a broad, general sense, and its relationships with health issues (Heinrich & Gullone, 2006). Studies among young adults have shown that they have existential thoughts about life and meaning, which are connected to negative experiences and emotions (Lundvall et al., 2020, 2019a). However, knowledge specifically about existential loneliness among adolescents is lacking (Mansfield et al., 2021).

Existential loneliness is described as one of the inevitable conditions of life, and a part of being human (Yalom, 1980). Studies that have focused on existential loneliness among adults in vulnerable situations (Bolmsjö et al., 2019; Ettema et al., 2010) show that it often arises in connection with transitions, when thoughts about meaning and meaninglessness come to the surface. Adolescence is a time of instability, when adolescents try to find meaning and to form their identity. They undergo both physical and relational changes in the transition between childhood and adulthood, and it is therefore likely that experiences of existential loneliness also occur among them. Adolescents have thoughts about life, existence and meaning, but do not always address them, or are not aware of them, as existential. The thoughts may lead to negative emotions and can even be shown through bodily expressions. According to the philosopher Merleau-Ponty, the body and mind are intertwined, and a person is a *lived body*, which simultaneously encompasses the physical, mental, and existential aspects of life (Merleau-Ponty & Landers, 2014), and emotions are formed and expressed in lived bodies, and occur and create meaning within social relationships (Burkitt, 2014). It is known that loneliness can show itself in bodily expressions such as pain, while at the same time it is a challenge for people in the sufferer’s environment to recognize, interpret and understand the underlying causes of these expressions. Therefore, it can be difficult to recognize existential loneliness and offer adequate support. There is an imminent risk that bodily expressions of everyday aspects of being human can be interpreted as pathological (Horwitz & Wakefield, 2007) and thus medicated, rather than met adequately. Health includes the whole human being and needs to be understood in relation to existence. When meeting adolescents it is therefore important to recognize, interpret and understand their existential life conditions and needs in order to promote their existential health and well-being and to prevent them from experiencing mental health problems. Therefore, the aim of this study was to explore adolescents’ experiences of existential loneliness.

## Methods

### Study design

This study had an exploratory-descriptive qualitative design. The data consisted of narrative research interviews (Kvale & Brinkmann, 2009), which were analysed inductively using qualitative conventional content analysis (Hsieh & Shannon, 2005). The study is reported according to The Consolidated Criteria for Reporting Qualitative Studies (COREQ) guidelines (Tong et al., 2007).

### Participants

The study employed purposive sampling, and the criteria for inclusion were adolescents attending upper secondary school or last year of compulsory school who had experienced existential loneliness. The intention was to obtain a varied sample and thus rich and varied data material (Creswell & Creswell, 2018), and therefore, both public and private schools in rural and urban areas in the south of Sweden were contacted by email. School nurses and teachers were informed about the study and were asked to be study facilitators. The study facilitators informed students about the study, and if interest was expressed in talking about experiences of existential loneliness, the study facilitators informed the first author, who contacted the students and provided information about the study orally and in writing. The students then signed informed consent forms and were encouraged to decide on the place and time for the interview. There is no consensus on which years adolescence encompasses, but in literature it is described as the phase between the ages of 10 and 24 years (Sawyer et al., 2018), and the ages 15 to 19 years are described as a phase of growth and consolidation (Bundy et al., 2017). In this study a total of 16 adolescents participated, aged 15 to 21 years (median age 17.5), comprising 10 persons who identified themselves as girls and six who identified themselves as boys. The adolescents were attending five different schools; two adolescents were in the last year of compulsory school, and 14 were students in private or public upper secondary schools who were attending both vocational and higher education preparatory programmes. Four adolescents who initially showed interest in participation declined for unknown reasons before the interviews were conducted. See, Table 1 for a description of the final sample.

**Table 1.** Characteristics of the sample.

Fictitious name	Age	Stated gender	School	Programme	School location area
Agnes	15	Girl	Public compulsory	General	Rural
Alexandra	15	Girl	Public compulsory	General	Rural
Alex	16	Boy	Private upper secondary	Vocational	Rural
Alfred	16	Boy	Private upper secondary	Higher education preparatory	Rural
Alma	16	Girl	Private upper secondary	Vocational	Urban
Alice	17	Girl	Public upper secondary	Vocational	Rural
Albin	17	Boy	Private upper secondary	Vocational	Rural
Annie	17	Girl	Private upper secondary	Vocational	Urban
Alva	18	Girl	Private upper secondary	Higher education preparatory	Urban
Amanda	18	Girl	Private upper secondary	Vocational	Rural
Anna	18	Girl	Public upper secondary	Vocational	Urban
Adrian	18	Boy	Private upper secondary	Higher education preparatory	Urban
Adam	19	Boy	Private upper secondary	Higher education preparatory	Urban
Andrea	19	Girl	Private upper secondary	Higher education preparatory	Urban
Alicia	19	Girl	Private upper secondary	Higher education preparatory	Urban
Ali	21	Boy	Public upper secondary	Vocational	Rural

### Data collection

The data were collected through individual narrative interviews carried out between September 2020 and September 2021 by the first author, who is a PhD student and experienced registered nurse. The open-ended questions have been used and tested in prior studies focusing on existential loneliness among frail older people (Edberg & Bolmsjö, 2019; Sjöberg et al., 2018, 2019). Two pilot interviews with adolescents were held to evaluate the questions, and no adaptions of the questions were considered necessary. The interview guide is available as a supplementary file. Since the pilot interviews responded to the aim of this study, and were considered to be of high quality when evaluated by the authors, they were included in this study.

The participants decided where the interviews should be carried out; 12 interviews were conducted using a digital meeting tool and four took place in a separate room at the participants’ schools. The rooms at the schools were chosen so privacy and confidentiality could be achieved. Those aspects were also in focus when the digital meeting tool was used; the researcher emphasized the importance of privacy and clarified that only the participant and the researcher should be present during the interviews. The adolescents chose to sit at home in privacy. Initial conversations were held to allow the participants and the researcher to become acquainted. To open the way for the question in focus, the researcher started the interview with an open-ended question about loneliness in general, and thereafter, the participants were asked to narrate their experiences of existential loneliness in particular. The question included a description of existential loneliness as a deep form of loneliness that can come and go. Additional probing questions were used to deepen the narratives. The interviews lasted a median of 50 minutes (range 24 to 86 minutes) and were digitally audio-recorded. After each interview, reflective field notes were taken. The first 11 interviews were transcribed verbatim by the first author and the last five by a secretary, with validation by the first author.

### Data analysis

The transcribed interviews were inductively analysed using qualitative conventional content analysis, as described by Hsieh and Shannon (2005). This approach is characterized by staying close to the text and allowing categories to emerge directly from the data. The analysis was completed manually. First, all four authors independently read all the texts to gain an overall understanding of the adolescents’ experiences of existential loneliness. The authors then discussed and compared their initial understanding of the text as a whole. Thereafter, meaning units related to the aim of the study were identified in the text, then coded, sorted and grouped into clusters by the first author, and discussed with the last author. Then all four authors reread the codes and meaning units and compared them, searching for differences and similarities which formed the basis for categories and subcategories. The meaning units of each subcategory were then brought together and treated as a new text that was condensed. The purpose was to obtain a narrative description of adolescents’ experiences of existential loneliness by using the words and narrations used by the adolescents themselves. To create an understanding of whether the results could be understood and related to being young, the results were also discussed with a reference group containing adolescents, that was recruited in connection to this study.

### Ethical considerations

The study was approved by the Ethical Review Agency in Sweden (EPN 2018:842; approved supplementary application 2020–01735), and all procedures were conducted in accordance with the Declaration of Helsinki (WMA, 2013). The study involved adolescents, who in general are seen as especially vulnerable persons; their participation in research is important in order to create understanding and allow their voices to be heard (Åkerström & Brunnberg, 2013; WMA, 2013). Therefore, the interviewer must be particularly responsive, especially when the research topic could bring up negative emotions (Crane & Broome, 2017). The interviewer strove to be sensitive and to respect the participants’ integrity and well-being. School health care personnel were informed in advance about the study, and the interviewer could mediate contact if needed. The voluntary nature of participation was also emphasized, which is especially important when the participants are recruited in the school environment (Connor et al., 2018). In the analysis, and when presenting the findings, the aim was to stay close to the text and use the adolescents’ own words and narrations with the purpose of allowing their voices to be heard.

## Findings

**Figure 1.**
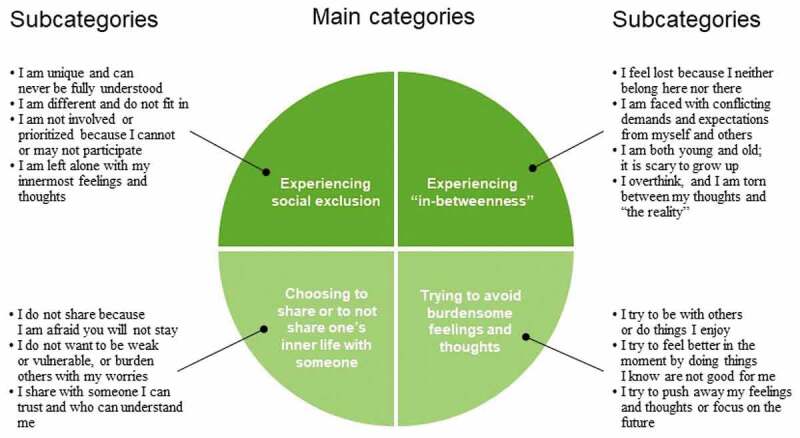
Main categories and subcategories of adolescents’ experiences of existential loneliness.

The adolescents’ experiences of existential loneliness are represented by four main categories, with internal variations in subcategories (see, Figure 1 for an overview of the main categories and subcategories). The main categories are: Experiencing social exclusion, Experiencing “in-betweenness”, Choosing to share or not share one’s inner life with someone, and Trying to avoid burdensome feelings and thoughts. The categories and subcategories are presented in the upcoming section in bold type. Participants’ quotes are reproduced in italics, and fictitious names are used.

### Experiencing social exclusion

Existential loneliness among adolescents was related to experiences of social exclusion, in the sense of not being involved or prioritized, being left alone with their innermost feelings and thoughts, being different and not fitting in, and being unique and not understood.

Insights about every person’s uniqueness and of never having the same experiences as anyone else, and therefore never being able to be fully understood, brought up deep feelings of loneliness among the adolescents. They could feel despair even when people were around. This was expressed as **I am unique and can never be fully understood**. According to the adolescents, nothing or nobody really mattered since humans were insignificant in the universe. Adam narrated experiences of *being completely separated from the rest of the world … and not being able to fend for [himself]*. The insights were connected to feelings of freedom, but mainly to those of being imprisoned in the uniqueness.

In addition, experiences of social exclusion were linked to a sense of not belonging because of being different from others, which was expressed as **I am different and do not fit in**. Experiences of never fitting in led to having fearful thoughts of being lonely for the rest of their lives. Adrian explained:

*That’s what you should call a* *kind of existential loneliness* *… You just think that this is a* *spiral that you will not be able to get out of* *… When I* *enter life, I* *will be alone* *… I* *had no literal nightmares, but it felt a* *bit like such* *… to kind of know that one will, like, not take part in life.*

However, experiences of difference could be positive in a sense of feeling better and more interesting than others. Despite these positive aspects, though, being different was still connected with feelings of loneliness and a sense of being “from another planet”. Feeling more mature and taking on more adult responsibility than friends took on meant not fitting in. This made it difficult to find common interests and topics of conversation, and the peers therefore gradually drifted apart.

The experience of social exclusion could occur regardless of whether it was self-selected or not. It was expressed as **I am not involved or prioritized because I cannot or may not participate**, which felt like being invisible. Alicia said that she has always been left out and de-prioritized because her younger siblings have special needs, and therefore she has had to stand aside. She described deep feelings of loneliness connected to *not being someone’s priority or most important to anyone*. Not having begun menstruation yet made it difficult to feel involved when peers talked about it, and it also raised worries concerning whether there was anything wrong with one’s own body. In addition, not being invited when peers hung out was painful at the time but also afterwards when they talked about all the fun they had had. However, sometimes it felt better to make the choice not to participate than to risk being the one who was not invited.

The adolescents thought it would have made a big difference to talk about their innermost feelings and thoughts with someone who really understood them. But experiences of being socially excluded and not being able to share their innermost feelings and thoughts with anyone was painful, and was expressed as **I am left alone with my innermost feelings and thoughts**. Not being able to share with one’s own mother was especially painful due to the adolescents’ assumptions that mothers are supposed to be interested and involved in their children’s lives. In addition, having experiences of never sharing with anyone made it difficult to suddenly start sharing. Experiences of losing a person they had previously shared with were described as losing the anchor. Alice said: *It feels like the whole world is going down*. To be left alone with the innermost feelings and thoughts felt empty, heavy, and deeply lonely.

### Experiencing “in-betweenness”

Existential loneliness among adolescents was also related to experiences of being in between, and therefore having a sense of not belonging anywhere, i.e., experiencing “in-betweenness”. It felt like being lost or being torn between different thoughts and “the reality”, and it was difficult being faced with conflicting demands and expectations, especially when the coming adulthood felt scary.

The adolescents who had been forced to leave their homelands explained that it was difficult to feel at home in their new country. The experiences during their escape meant personal growth, which led to feelings of no longer belonging in the former homeland either. The “in-betweenness” was expressed as **I feel lost because I neither belong here nor there**. Fears of having to return to the homeland or not finding their place in the new country was therefore painful. Even for those born in Sweden, feelings of being lost and in between occurred. When parents had a different cultural background, their values did not always agree with Swedish values, and it was difficult to know how to behave and where to belong. For Amanda, the sense of “in-betweenness” emerged when she dropped out of school and started an internship. She stated:

*You felt that you ended up in between* *… You did not really feel like a* *part of anything at all. So, you were, like, in a* *kind of intermediate position* *… You feel very lost anyway.*

She did not feel part of her peer-group because she no longer hung out with them at school, and at the same time she was not part of the work community where she was carrying out her internship because she was there in the role of intern and not of co-worker.

Furthermore, being faced with conflicting demands and expectations made it difficult to know which way to turn. The adolescents’ own and others’ expectations of constant development and achievement led to perceptions of never really being good enough and fears of never, ever succeeding. When pressured by both peers and parents, whose expectations were conflicting, experiences of “in-betweenness” and being torn and in the middle arose. They were expressed as **I am faced with conflicting demands and expectations from myself and others**. Andrea explained, *You do not manage, but you have to, and you kind of do not understand how to solve everything*. In addition, expectations of adolescence as a period in life when one is supposed to have fun felt overwhelming when everyday necessities were already difficult to live up to.

It was hard to feel both young and old, and sometimes wanting to lie in their mothers’ arms and be comforted, and at other times to be independent and manage on their own. Becoming an adult was scary because it meant increased responsibility and a great deal of uncertainty about the future. This was expressed as **I am both young and old; it is scary to grow up**. Alva said, *I am still trying to figure out what it means to be an adult*. She feared that the whole world as she knew it would be turned upside down after her 18th birthday. She also knew that she would lose her many years of contact with child and adolescent psychiatry. All in all, the experiences were scary, and suicidal thoughts occurred that at first felt liberating because they meant she would not have to grow up, but at the same time brought feelings of hopelessness and of being stuck because she did not want to inflict suffering on her family.

Getting stuck in painful and worrying thoughts through overthinking was related to experiencing “in-betweenness”. The negative thoughts were about the participants’ own appearance and others’ perceptions, about not being good enough or about fears of being abandoned. Although the thoughts did not correspond to “reality”, it was difficult to let them go, and experiences of being torn between the thoughts and “the reality” arose, which led to worrying feelings and a sense of being deeply alone with these experiences. This was expressed as **I overthink, and I am torn between my thoughts and “the reality”**. Adrian narrated his difficulties in leaving what he described as *an emotional prison* that he had created himself, which prevented him from trusting other people and building relationships. When eventually he focused on the realization that these thoughts were untrue, it meant that he could influence his situation for the better, which was felt to be a relief.

### Choosing to share or not to share one’s inner life with someone

Choosing to share their inner lives with someone could be a way to alleviate existential loneliness, though far from all adolescents chose to share. Those who chose to share emphasized the importance of trust and being understood. Choosing not to share, on the other hand, was related to not wanting to be seen as weak and vulnerable, wanting to avoid burdening others with worries, or having fears of losing relationships.

The adolescents had friends, but often no-one with whom they chose to share their inner lives with. They were afraid that if their peers were to know the truth about them, they would be abandoned, or that the relationship should change, which might lead to not being able to have fun together again. In addition, previous negative experiences of being abandoned or betrayed made them keep others at a distance. The experiences were expressed as **I do not share because I am afraid you will not stay**, which made them feel empty and deeply lonely. Choices not to share were also connected to being obliged to change schools constantly, which led to experiences of deep loneliness. Anna said, *I am left to myself and my own thoughts and feelings*. She said that she had stopped sharing her inner life with others because she knew that she risked losing the relationships she had built. She had been through this several times already due to constant moves which she could not influence.

The adolescents reasoned that showing feelings was connected to being vulnerable. By “wearing a mask” they did not show weakness, and in that way the inner self could be hidden. Thoughts about others seeing them as happy people prevented them from sharing and showing who they really were, and they did not want to see themselves as people with worrying feelings. The choice not to share with anyone was also about not wanting to burden others with worries, and the realization that others might feel worse than they did. Therefore, the adolescents chose to keep their worries to themselves, and no-one really knew them on a deeper level. The experiences were expressed as **I do not want to be weak or vulnerable, or burden others with my worries**. Alexandra said, *It makes me very, very lonely with my feelings*. Aspects of bonding with another human being also felt uncomfortable. However, not sharing meant that no-one else shared either, which made it difficult to determine which feelings were normal. In addition, it could be a boost to their self-confidence if they were able to manage on their own. Therefore, sharing, which implied asking for help, was not an option.

The adolescents who had shared their inner lives with someone emphasized the importance of trust and understanding. This was expressed as **I share with someone I can trust and who can understand me**. Sharing with their closest friends meant being able to obtain support without needing to give an explanation when life sometimes felt difficult. When peers were not fully trusted, it felt better to share with a girlfriend, boyfriend, or mother. The adolescents also had positive experiences of talking to professionals such as a counsellor, teacher, or psychologist, who were adults they could trust. However, sometimes it was better to talk to a friend of the same age. Alex explained that he preferred to share existential thoughts and feelings with his friends. When he had tried to talk to adults, he experienced that they already had ready-made answers, even though he just wanted to discuss his thoughts. He said:

*Then they have already thought about it and figured it out. It helped me absolutely not at all.*

### Trying to avoid burdensome feelings and thoughts

To alleviate existential loneliness the adolescents tried to avoid burdensome feelings and thoughts. They tried to feel better in the moment by doing things they knew were not good for them. They also tried to push away their feelings and thoughts and focus on the future instead of a burdensome present or past. In addition, they tried to spend time with others or do things they enjoyed.

Hanging out with family or friends meant that the burdensome feelings and thoughts did not become so intrusive. Participating indirectly via social media when not participating with peers “in real life” could decrease feelings of deep loneliness, and it sometimes felt easier to make contact with peers on the internet. The adolescents also tried to do things they enjoyed to help them to avoid burdensome feelings and thoughts. The experiences were expressed as **I try to be with others or do things I enjoy**. Since they all had different interests, they chose to do various things such as listening to music, singing, watching TV, playing computer and video games, hanging out in the stable, driving a tractor or going running. These activities created a break from burdensome feelings and thoughts and provided moments of peace and well-being. One participant, Ali, had forced himself out of his isolation on one occasion when he was a newcomer in Sweden and doubted whether he would have been alive today if he had not gone out that day and run into a person who knew his language. He described that new acquaintance as *a rescuer*, since the suffering connected to the isolation and the deep feelings of loneliness was extensive and unbearable.

Substances and destructive thoughts were sometimes used in the moment by the adolescents to get away from burdensome feelings, even though they were aware that these were not good for them in the long term. This was expressed as **I try to feel better in the moment by doing things I know are not good for me**. Alva said that tobacco and alcohol were her *self-medication and comfort*, which had unfortunately led her into an addiction that she did not know how to get out of. She sometimes thought about her own death to feel relief, because being dead would mean that the burdensome feelings would disappear. Thinking about non-suicidal self-injury was also a way of avoiding the burdensome thoughts and feeling relief. However, realizing the thoughts as actions would have led to negative consequences and feelings of losing themselves, and was therefore avoided.

To avoid burdensome feelings and thoughts, the adolescents sometimes tried to push away their feelings and thoughts. By focusing on the future, they tried to avoid thinking about a burdensome present or past, with the aim of reducing existential loneliness as well. This was expressed as **I try to push away my feelings and thoughts or focus on the future**. Adam described himself as an expert on pushing away feelings and thoughts, even though it did not feel a good thing to do. He said:

*[I] showed feelings as a* *little boy when the society says that boys should show fewer feelings. Then there has been a* *bit of a* *clash there, and then one has probably tried to adapt to it, and then it has become extreme in the other direction* *… I* *find it quite easy to push away feelings.*

When the adolescents were trying to push away deep feelings of loneliness, the feelings did not disappear but instead became deeper.

## Discussion

We have explored adolescents’ experiences of existential loneliness. The core findings of the study showed that existential loneliness among adolescents is related to experiencing social exclusion and “in-betweenness”. To alleviate their suffering, the adolescents sometimes tried to avoid burdensome feelings and thoughts, and they chose between sharing or not sharing their inner lives with someone. They wanted to be listened to and understood by someone they could trust. Existential loneliness, which is described as a part of being human, comprised primarily negative experiences among adolescents, which is in line with previous research among adults (Mansfield et al., 2021).

We found that existential loneliness among adolescents is connected to experiencing social exclusion and “in-betweenness”; experiences related to a deep sense of not belonging. Humans have an inner desire to belong and to feel connected with others (Yuval-Davis, 2006). Belongingness arises from the interaction between the individual, other people, and socio-cultural contexts (Halse, 2018), and is dependent on power structures (Yuval-Davis, 2006) and social norms (Wright, 2015). Social norms are important in the formation of identity (McDonald & Crandall, 2015) and thus crucial during adolescence. The term “in-betweenness” has previously primarily been used in studies on transculturality, where it is described as a sense of “double exclusion” that influences the shaping of identity (Kılınç et al., 2022). When trying to form the identity during the transition between childhood and adulthood, the adolescents in our study were experiencing an unfamiliar situation, which sometimes made them feel deeply lonely. They occasionally experienced being completely separated from the rest of the world, and they described the present as sometimes hard to deal with and their future adulthood as uncertain and scary. Their experiences have similarities with adults’ experiences. In studies among older people, the meaning of existential loneliness has been expressed as being disconnected from life (Sjöberg et al., 2018) and as being in transition, navigating an unfamiliar situation, longing for togetherness, and being excluded (Larsson et al., 2020). Previous studies focusing on social exclusion and not belonging have shown relationships with pain (Bernstein & Claypool, 2012), loneliness (Rönkä et al., 2018), depressive symptoms (Parr et al., 2020) and mental illness (Arslan, 2020; Santini et al., 2021). Belongingness, on the other hand, has been shown to be a protective factor against loneliness (Baskin et al., 2010) and a facilitator in adolescents’ transition into adulthood (Tanti et al., 2011). School is a critical arena for adolescents’ development (García-Moya et al., 2019), and school belongingness or connectedness has been shown to promote mental health and well-being (Lester et al., 2013) as well as psychosocial functioning and academic performance (Allen et al., 2018). Previous research shows that important factors to alleviate loneliness among adolescents are interventions focusing on strengthening the self, working on safe environments, and inclusion (Sundqvist & Hemberg, 2021). Thus, it is important to support adolescents’ sense of inclusion and belonging to promote their health and well-being.

In our study the adolescents tried to ease their suffering by trying to avoid burdensome feelings and thoughts, even if it meant doing things they knew were not good for them. Previous studies have shown connections between loneliness and health-risk behaviours such as substance use among adolescents (Stickley et al., 2014). Our study also showed other ways of handling existential loneliness, such as changing focus, doing enjoyable things, and spending time with others. This is in line with studies focusing on existential loneliness among frail older people, where it has been shown to be eased by experiencing meaningful togetherness with others, and focusing on other things in order to put existential loneliness into the background (Sjöberg et al., 2019). Changing focus and putting the suffering into the background can be understood through the Shifting Perspectives Model of Chronic Illness (Paterson, 2001). The model reflects individual needs and emphasizes the importance of being aware of the dialectical and constantly shifting perspectives where either wellness or illness is in the foreground or the background. By allowing the suffering to be in the background, restful spaces are created, and by allowing it from time to time to be in the foreground, the experiences may be shared with somebody. The adolescents in our study tried to ease their suffering in a variety of different ways, which can be interpreted as trying to rest from the suffering for a while by putting their existential loneliness into the background. However, they also addressed the need to share their inner thoughts and feelings with somebody, which can be interpreted as allowing the suffering to be in the foreground. Thus, adolescents may need assistance in finding constructive and healthy ways to rest from the suffering, as well as support in dealing with experiences of existential loneliness in safe and trustful environments.

However, sharing one’s inner life with another person is not always easy. Those in our study who chose not to share with someone were afraid of losing relationships, and they did not want to overburden others with their worries or risk being seen as weak or vulnerable. Previous studies have shown loneliness to be linked to shame and a desire not to overburden others (Hemberg et al., 2021). Studies of existential concerns among young adults have shown that they suffered in silence because they did not want to be perceived as vulnerable, they were afraid of being rejected if they shared, and they were longing to share their inner thoughts with someone whom they could trust (Lundvall et al., 2020, 2019a), which is in line with our results. When deviating from social norms there is a risk of being socially excluded. Therefore, it may feel safer not to share one’s innermost feelings and thoughts with anyone, rather than risk being seen as abnormal. Trust is an important factor when choosing to share, which was shown in our study. Sharing with a trusted adult who listens and who understands may be preferable to risking being socially excluded by peers. In previous research it has been shown that existential loneliness can even lead to personal development and growth when it is acknowledged and addressed (Ettema et al., 2010), and sharing may therefore be important when experiencing such loneliness. Adolescents who feel emotionally supported and have access to people who can assist them have also been shown to have a better chance of managing stressful situations and adapting to new environments (Khawaja et al., 2018). However, existential loneliness may be difficult to recognize, since it is not always obvious. In previous studies it has been shown that nursing staff and volunteers found it challenging to recognize and meet older people’s existential loneliness (Sundström et al., 2021, 2018). In addition, health care professionals found it challenging to deal with young adults’ existential concerns, and they needed support to create an inviting atmosphere and stay present, since the conversations could stir up their own existential issues (Lundvall et al., 2019b). Thus, professionals need knowledge and an understanding of existential loneliness to be able to identify and meet it adequately. They also need courage and support. By approaching their own existential needs, and having frequent discussions with colleagues about existential issues, opportunities for self-awareness and understanding are created. This may be helpful when trying to meet adolescents’ existential needs to promote their existential health and well-being.

### Strengths and limitations

A challenge of research among adolescents is the negative impact of power structures between the adolescents and the adult researchers, which may be a hinderance, especially when focusing on emotional aspects. The researchers in this study are middle-aged, privileged women and therefore, the adolescents could have perceived themselves as disadvantaged during the interviews. The researchers, on the other hand, risked having difficulties in understanding the adolescents during the analysis. Therefore, firstly by allowing the adolescents to choose where the interviews should be conducted and by conducting the interviews using a person-centred approach, the aim was to reduce the negative impact of those existing power structures and to create environments that were as safe and trustful as possible. An empathetic conversational climate was important, and the first author, who conducted the interviews, is an experienced registered nurse specializing in psychiatric nursing, familiar with interview techniques and with solid experience of talking to people about existential issues. In addition, among the authors there are experienced qualitative researchers who are familiar with research among adolescents, which was a strength when interpreting the results. However, to reduce the influence of researcher bias, and to allow the adolescents’ voices to be heard and understood, we stayed close to the text during the analysis and used a reference group containing adolescents to discuss the findings.

Furthermore, several interviews were conducted digitally, which can have both pros and cons. The digital format can lead to difficulties in reading body language, for example, but on the other hand, it can be perceived positively by the adolescents as an opportunity to talk about emotional aspects without having to meet. In this study the interview text was rich with a variety of experiences of existential loneliness, which can be interpreted as an indication of good quality in the interviews regardless of the format in which they were conducted. In addition, the interview questions have been used in previous studies (Edberg, 2021; Edberg & Bolmsjö, 2019) and were also tested through pilot interviews, which is a strength in this study. The variation in the sample also increases the possibility of transferring the findings. However, as all the adolescents in this study were attending school, the results may not be transferrable to adolescents who are outside the school system.

Another challenge of research among adolescents is that they are young and thus particularly vulnerable. Ethical considerations were therefore borne in mind during the entire research process, and the study’s approval by the Ethical Review Agency in Sweden was a prerequisite for the implementation of the study.

## Conclusion

Adolescents experience existential loneliness in the transition between childhood and adulthood. Existential loneliness should not be seen as pathological, but as part of being human. It is related to suffering but may lead to positive outcomes if it is acknowledged and addressed. It is important to support adolescents’ sense of belonging to promote their health and well-being, and the adolescents may need assistance in finding constructive and healthy ways of resting from the suffering, as well as support in meeting existential loneliness in safe and trustful environments. Also, having the opportunity to talk about existential loneliness with someone who understands is important. However, experiences of existential loneliness are difficult to recognize and deal with adequately, and they can also bring up existential issues for the listener. Therefore, professionals who meet with adolescents need support, knowledge and understanding of existential loneliness to promote the adolescents’ existential health and well-being. Further research is needed that deepens the understanding of existential loneliness during adolescence, as well as the understanding of professionals’ experiences of promoting adolescents’ existential health.

## Data Availability

The data presented in this study are available on request from the corresponding author : tide.garnow@hkr.se. The data are not yet publicly available due to work in progress.
